# Analyses of nicotine metabolism biomarker genetics stratified by sex in African and European Americans

**DOI:** 10.1038/s41598-021-98883-z

**Published:** 2021-10-01

**Authors:** Meghan J. Chenoweth, Lisa Sanderson Cox, Nikki L. Nollen, Jasjit S. Ahluwalia, Neal L. Benowitz, Caryn Lerman, Jo Knight, Rachel F. Tyndale

**Affiliations:** 1 Campbell Family Mental Health Research Institute, Centre for Addiction and Mental Health, Toronto, ON Canada; 2 Department of Pharmacology and Toxicology, University of Toronto, Toronto, ON Canada; 3 Department of Population Health, University of Kansas School of Medicine, Kansas City, KS USA; 4 Departments of Behavioral and Social Sciences and Medicine, Brown University, Providence, Rhode Island USA; 5 Department of Medicine, University of California, San Francisco, San Francisco, CA USA; 6 USC Norris Comprehensive Cancer Center, Keck School of Medicine, University of Southern California, Los Angeles, CA USA; 7 Data Science Institute and Lancaster University Medical School, Lancaster, UK; 8 Department of Psychiatry, University of Toronto, Toronto, ON Canada; 9 Department of Pharmacology and Toxicology, University of Toronto, Medical Sciences Building Room 4326, 1 King’s College Circle, Toronto, ON M5S 1A8 Canada

**Keywords:** Genetics, Neuroscience, Biomarkers

## Abstract

Nicotine is inactivated by the polymorphic CYP2A6 enzyme to cotinine and then to 3′hydroxycotinine. The Nicotine Metabolite Ratio (NMR; 3′hydroxycotinine/cotinine) is a heritable nicotine metabolism biomarker, varies with sex and ancestry, and influences smoking cessation and disease risk. We conducted sex-stratified genome-wide association studies of the NMR in European American (EA) and African American (AA) smokers (NCT01314001, NCT00666978). In EA females (n = 389) and males (n = 541), one significant (P < 5e−8) chromosome 19 locus was found (top variant: rs56113850, *CYP2A6* (intronic), for C vs. T: females: beta = 0.67, P = 7.5e−22, 21.8% variation explained; males: beta = 0.75, P = 1.2e−37, 26.1% variation explained). In AA females (n = 503) and males (n = 352), the top variant was found on chromosome 19 but differed by sex (females: rs11878604, *CYP2A6* (~ 16 kb 3′), for C vs. T: beta = − 0.71, P = 6.6e−26, 16.2% variation explained; males: rs3865454, *CYP2A6* (~ 7 kb 3′), for G vs. T: beta = 0.64, P = 1.9e−19, 18.9% variation explained). In AA females, a significant region was found on chromosome 12 (top variant: rs12425845: P = 5.0e−9, *TMEM132C* (~ 1 Mb 5′), 6.1% variation explained) which was not significant in AA males. In AA males, significant regions were found on chromosomes 6 (top variant: rs9379805: P = 4.8e−9, *SLC17A2* (~ 8 kb 5′), 8.0% variation explained) and 16 (top variant: rs77368288: P = 3.5e−8, *ZNF469* (~ 92 kb 5′), 7.1% variation explained) which were not significant in AA females. Further investigation of these associations outside of chromosome 19 is required, as they did not replicate. Understanding how sex and ancestry influence nicotine metabolism genetics may improve personalized approaches for smoking cessation and risk prediction for tobacco-related diseases.

## Introduction

Cigarette smoking remains a major public health concern^1^. Smoking behaviors, including the response to pharmacotherapy, differ between men and women. For instance, varenicline is more efficacious compared to nicotine patch or bupropion for women, whereas the treatment efficacy for all three is similar for men^2^. Smoking cessation is influenced by genetic variation in *CYP2A6*, coding for CYP2A6 which inactivates nicotine to cotinine, and metabolizes cotinine to 3′hydroxycotinine^3,4^. The nicotine metabolite ratio (NMR; 3′hydroxycotinine/cotinine ratio) is a biomarker of CYP2A6 activity, with higher NMR reflecting faster nicotine clearance^5^. CYP2A6 is induced by estrogen^6^, thus post-pubertal females who are pre-menopausal have faster nicotine clearance^7^ and higher NMR^8^ compared to males. African American (AA) smokers have lower NMR compared to European American (EA) smokers, due in part to AA having a higher prevalence of *CYP2A6* decreased or loss-of-function variants^9^ and potentially also due to a higher prevalence of mentholated cigarette smoking^8^ compared to EA. Menthol can modestly inhibit CYP2A6 activity in vitro^10^ and nicotine clearance in vivo^11^, and smokers of mentholated cigarettes have lower NMRs on average^8^. Thus, sex, ancestry, and potentially mentholated cigarette smoking can alter CYP2A6 activity and the NMR.

The NMR has been associated with treatment response in bupropion and nicotine patch trials^12–14^. In the Pharmacogenetics of Nicotine Addiction Treatment (PNAT)-2 clinical trial (NCT01314001) where smokers were prospectively randomized by pre-treatment NMR, those with higher NMR had superior quit rates on varenicline (vs. nicotine patch), whereas those with lower NMR had similar quit rates^15^. Higher (vs. lower) CYP2A6 activity (measured by the NMR or *CYP2A6* genetics) is also associated with higher exposure to tobacco-specific nitrosamines, volatile organic compounds, polycyclic aromatic hydrocarbons, and inflammatory markers^16^ and higher risk for tobacco-related diseases such as lung cancer^17–19^. *CYP2A6* variation also alters the metabolism of chemotherapeutic agents (e.g. tegafur^20^ and letrozole^21^). In contrast to genomic assessments, the NMR can be reliably obtained only in current tobacco users^22^. An increased understanding of the genetics of the NMR, and if they differ by sex and ancestry, will enhance application in oncology and in epidemiologic studies of disease risk where non- and former-smokers are included.

Heritability estimates for the NMR range from 60 to 80%^23,24^. The vast majority of genome-wide significant (GWS) variants found in genome-wide association studies (GWASs) of the NMR in smokers were found in the *CYP2A6* region of chromosome 19^24–28^; these GWASs all controlled for sex. Controlling for sex can confound the discovery and interpretation of genetic signals if the variant displays a sex-specific effect, or has a large but opposing direction of effect in males and females^29^. Sex differences in genetic effects on the expression and activity of CYP3A4, another CYP enzyme, have been reported^30^. Our primary aim was to identify sex differences in the genetic influences on CYP2A6 activity, measured by the NMR, and contrast these differences between AA and EA smokers. Compared to EA, far less is known regarding the biological underpinning of traits in minority populations including AA which have historically comprised < 5% of GWAS samples^31^. Our prior GWAS of the NMR (controlling for sex) in AA smokers identified several novel GWS SNPs in comparison to those found in EA smokers^26^. Here we performed sex-stratified GWASs of the NMR separately in EA and AA smokers.

## Methods

### Study participants

#### PNAT2 (NCT01314001) and Kick-it-at-Swope (KIS)-3 (NCT00666978) clinical trials

Genetic and biomarker data from PNAT2 and KIS3 clinical trial participants who provided written informed consent for DNA collection and release of de-identified information to investigators were used for this analysis, as previously described^26^. The clinical trial procedures were approved by IRBs at the University of Toronto and all clinical sites^15,32^ and were carried out in accordance with the Declaration of Helsinki. PNAT2 participants were EA and AA adult smokers (≥ 10 cigarettes/day) recruited at four clinical sites (University of Pennsylvania, University of Toronto/CAMH, SUNY Buffalo, and MD Anderson Cancer Center) and were randomized to placebo, nicotine patch, or varenicline based on their NMR at intake (for details see Ref.^15^). KIS3 participants were AA adult smokers from the Kansas City, Missouri area screened for participation in a clinical trial evaluating bupropion efficacy in AA light smokers (≤ 10 cigarettes/day) (for details see Ref.^32^).

### Genome-wide SNP genotyping, quality control procedures, and imputation

Genome-wide genotyping was conducted at the Centre for Applied Genomics (Hospital for Sick Children; Toronto, Canada) using the Illumina HumanOmniExpressExome-8 v1.2 array and an iSelect custom add-on comprising 2688 variants associated with nicotine metabolism and/or smoking behaviours^26^. Quality control (QC) procedures implemented prior to imputation included removing samples with discordant sex, call rates < 98%, relatedness PI_HAT > 0.185, and heterozygosity > 3SD outside the mean. Variants with call rates < 98%, Hardy–Weinberg Equilibrium P < 1e−6, and minor allele frequencies (MAF) < 1% were excluded. Full details are described elsewhere^26^. EA and AA ancestry was determined via multidimensional scaling in combination with data and visualization from HapMap 3 (as described^26^). Genetic ancestries were highly concordant (> 95%) with self-reported ancestries. The number of individuals and variants available for phasing and imputation was: 390 female and 545 male PNAT2 EAs, 667,810 variants; 255 female and 251 male PNAT2 AAs, 733,629 variants; and 304 female and 154 male KIS3 AAs, 742,493 variants. The Michigan Imputation Server^33^ imputed genotypes using ShapeIT v2.r790^34^ and the HRC version r1.1 and 1000G Phase 3 v5 reference panels for EA and AA, respectively. Post-imputation variants with info (i.e. imputation quality) scores > 0.60 and MAF > 1% were analyzed. A total of 7,602,147 and 7,599,895 variants were analyzed in EA females and EA males, respectively. A total of 17,199,451 and 17,379,949 variants were meta-analyzed in AA females and AA males, respectively.

### Phenotype: the nicotine metabolite ratio

As described elsewhere^26^, cotinine and 3′hydroxycotinine were measured using LC–MS/MS in PNAT2 and KIS3 participant blood samples collected at baseline during ad libitum smoking. To ensure reliability of NMR results, participants with cotinine < 10 ng/ml, suggestive of non-daily smoking, were excluded^26^. Seven PNAT2 participants (n = 5 EA and n = 2 AA) were excluded from further analyses due to missing and outlying (> 4 SD from the mean) NMR data, while n = 8 AA participants from KIS3 were excluded due to low cotinine. To correct for positive skew, the NMR (3′hydroxycotinine/cotinine) was rank-transformed using the rntransform function (https://rdrr.io/cran/GenABEL/man/rntransform.html) in the GenABEL package in RStudio.

### Sex- and ancestry-stratified GWAS of the NMR

Frequentist additive models in SNPTEST (version 2.5.2)^35^ identified genetic associations with rank-transformed NMR separately in PNAT2 EA females, PNAT2 EA males, PNAT2 AA females, PNAT2 AA males, KIS3 AA females, and KIS3 AA males. As is convention in SNPTEST, the outcome measure was mean centered and scaled to have a variance of 1 for analysis. Chromosomes 1–22 were analyzed. Genotype uncertainty was controlled for by using the “-method expected” option^26^. Principal components 1–4, calculated separately in PNAT2 EA, PNAT2 AA, and KIS3 AA, were included in all models to adjust for population substructure. In previous genomic investigations of the NMR in these datasets, controlling for the first 2 versus 4 PCs^26^, or up to 10 PCs^36^, did not substantially alter the results. Potential covariates were evaluated based on a priori variables associated with rank NMR in PNAT2^8^, which was the larger of the two trials and included both EA and AA. In EA, the main model included age (beta = 0.12; P = 0.0003), BMI (beta = − 0.10; P = 0.003), and alcohol use (# of drinks/week: beta = 0.06; P = 0.08) as covariates. In AA, the main model included age (beta = 0.12; P = 0.01), BMI (beta = − 0.10; P = 0.03), and mentholated cigarette use (beta = − 0.10; P = 0.047) as covariates. The final sample size for the main analysis was: n = 389 female and n = 541 male PNAT2 EA smokers, n = 205 female and n = 201 male PNAT2 AA smokers, and n = 298 female and n = 151 male KIS3 AA smokers. In a secondary analysis in EA, we further controlled for mentholated cigarette use. In a secondary analysis in KIS3 AA, where alcohol use (drinks/week) was significantly associated with NMR (beta = 0.16; P = 0.001), we further controlled for alcohol use. In PNAT2 AA, n = 98 participants were missing menthol data^26^, and were excluded from the main analysis; thus, we also performed a secondary analysis in all AA participants, without controlling for menthol. In KIS3 AA, n = 1 participant was missing BMI data, and was excluded from all analyses.

QQ plots are shown in Supplementary Fig. S1. Results from the PNAT2 AA females and KIS3 AA females were meta-analyzed, as were the results from the PNAT2 AA males and KIS3 AA males, using a fixed-effects model, info score threshold of 0.6, and META (version 1.7) software^37^.

To detect genome-wide association of individual SNPs explaining ≥ 10% of rank-transformed NMR variance, power was calculated to be 74%, 83%, 97%, and 98%, respectively, for sample sizes of n = 352, n = 389, n = 503, and n = 541. Notably, there was ≥ 80% power to detect SNPs explaining > 7% of rank-transformed NMR variation for n > 500. Power calculations were performed using Quanto version 1.2.4^38^ and considered an additive model, two-sided significance level of 5e-8, and 5% MAF.

### Conditional analysis, proportion of NMR variation explained, and sex × genotype interaction testing

To identify putative independent GWAS signals, conditional analyses were run in SNPTEST (version 2.5.2)^35^ for chromosomes that contained > 1 GWS variant, as previously described^26^. Analyses were performed separately for each chromosome, sex, and ancestry. For the AA sample, sex-stratified conditional analyses were performed in PNAT2 AA and KIS3 AA, followed by within sex meta-analysis. In conditional analysis, after controlling for the top variant, the next variant with the smallest P-value is controlled for along with the top independent signal in a second round of conditional analyses. The procedure is repeated until no additional significant (at P < 5e−8) signals emerge^26^.

In separate linear regression models examining the influence of genotype (coded additively: 0 vs. 1 vs. 2 copies of the effect allele) on rank-transformed NMR, we calculated the proportion of variation explained by each SNP by squaring the part correlation coefficient and multiplying by 100, after controlling for principal components 1–4 and NMR covariates (age, BMI, and alcohol use in EA; age, BMI, and mentholated cigarette smoking in AA). Analyses were conducted using SPSS version 23 (IBM, Armonk, New York, USA).

In AA, where sex-specific signals were observed, linear regression modeling examined the effect of the sex × genotype interaction term on rank-transformed NMR after controlling for NMR covariates (age, BMI, and menthol smoking) and main effects (sex and genotype, coded additively). Separate models were run for rs12425845, rs9379805, and rs77368288. Analyses were conducted using SPSS version 23 (IBM, Armonk, New York, USA).

### Testing for replication of sex-specific genetic influences on the NMR in AA smokers

To determine whether the sex-specific signals on chromosome 12 (in AA females) and on chromosomes 6 and 16 (in AA males) replicated in an external sample, AA smokers with NMR data from the Quit-2-Live (NCT01836276)^39^ (n = 108 females and n = 108 males) and KIS2^40^ (n = 334 females and n = 146 males) trials were analyzed. We genotyped samples for rs12426751 on chromosome 12, rs9295678 on chromosome 6, and rs77368288 on chromosome 16 using validated TaqMan SNP genotyping assays according to the manufacturer’s protocol (Applied Biosystems, Waltham, Massachusetts, USA). The rs12426751 and rs9295678 SNPs were selected since they are in high linkage disequilibrium (r^2^ = 0.95 in 1000G phase 3 AFR sample) with the sentinel SNPs (rs12425845 in chromosome 12 and rs9379805 in chromosome 6, respectively) and were directly genotyped by the array. For chromosome 16, the top SNP (rs77368288) was tested for replication.

The NMR was measured in KIS2 and Quit-2-Live at baseline during ad libitum smoking^39,41^. Linear regression models tested for association between genotype (coded additively) and rank-transformed NMR. Covariates included age, BMI, and mentholated cigarette smoking, which had similar distributions in the replication datasets (KIS2 and Quit-2-Live) as in the discovery datasets (PNAT2 and KIS3). Analyses were conducted using SPSS version 23 (IBM, Armonk, New York, USA). Assuming an additive model, alpha level of 0.05 for replication, and a similar MAF and effect size as found for the top chromosome 12, 6, and 16 SNPs in the discovery set, the power to detect an association in the replication set was > 0.99. Power was calculated using Quanto version 1.2.4^38^.

### eQTL and meQTL analysis

The GTEx Portal (https://www.gtexportal.org)^42^ and meQTL Database (http://www.mqtldb.org/)^43^ were used to determine whether the GWS variants were expression quantitative trait loci (eQTL) and/or methylation quantitative trait loci (meQTL), respectively. In GTEx, significant eQTL (using data release V8) were determined following cis-eQTL mapping using FastQTL, as described (https://www.gtexportal.org/home/documentationPage). For the meQTL analysis, the MatrixEQTL database and all time-points were selected, and the trans meQTL distance was set at > 1 Mb; i.e. variants found > 1 Mb away from the altered CpG position were defined as trans meQTL.

## Results

### Sex- and ancestry-stratified analysis of the NMR

**Figure 1:**
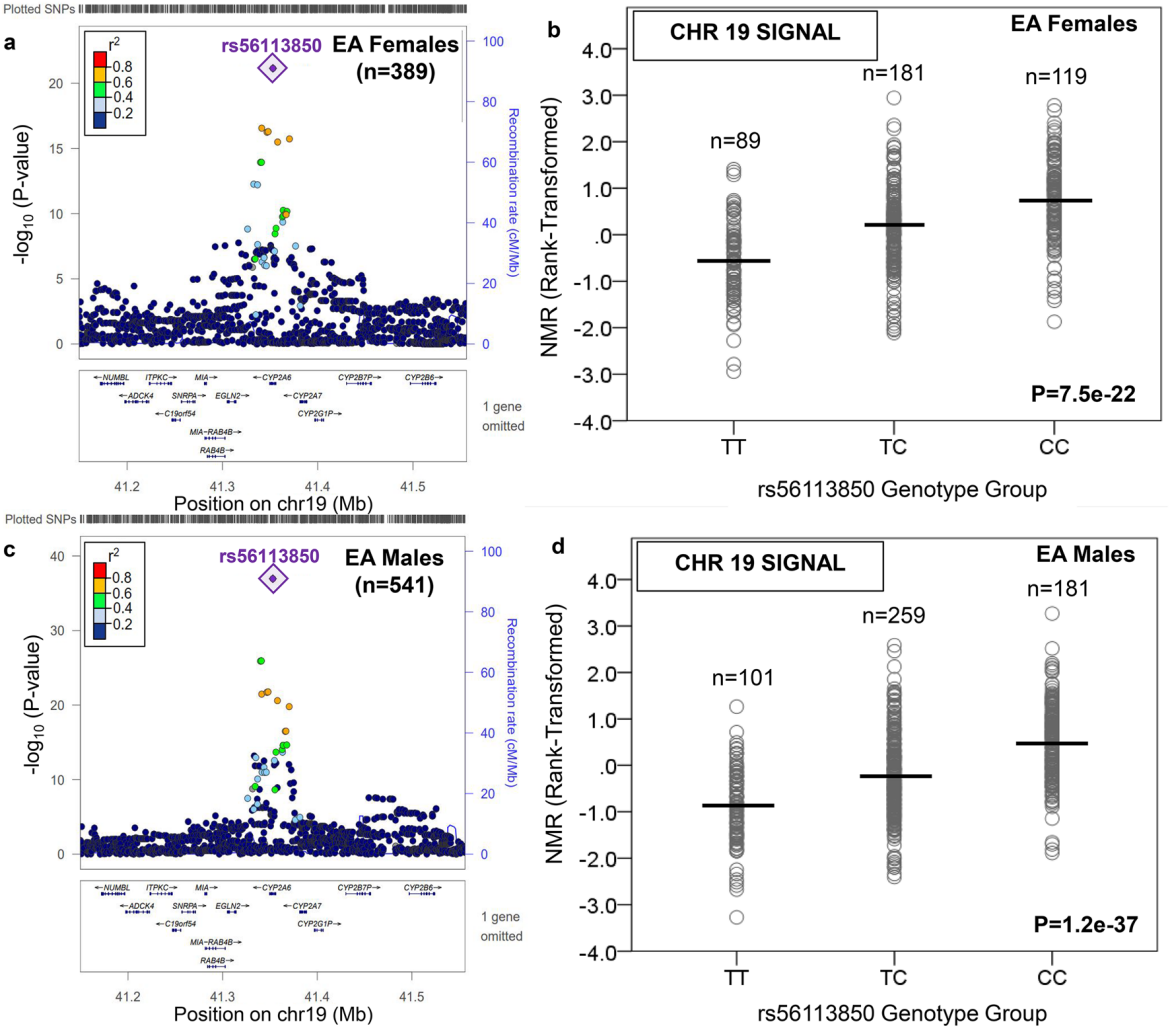
The top signal associated with the Nicotine Metabolite Ratio in European ancestry females and males was found on chromosome 19 in the region containing *CYP2A6*. The nicotine metabolite ratio was rank-transformed for analysis. Regional plots, generated using LocusZoom^44^ (freely available at locuszoom.org), show the top overall SNP in European American (EA) females (rs56113850) (**a**). The influence of rs56113850 on the Nicotine Metabolite Ratio (NMR) in EA females is shown in (**b**). The rs56113850 SNP was also the top SNP in EA males (**c**), and the influence of rs56113850 on the NMR in EA males is shown in **d**). Linkage disequilibrium patterns in (**a**) and (**c**) are based upon the hg19/1000 Genomes November 2014 release European reference population. The plots in (**b**) and (**d**) were created using SPSS version 23 (can be purchased from IBM, Armonk, New York, USA). The black horizontal line represents the mean rank-transformed NMR in each group, and the P-value is from the GWAS after adjusting for population substructure and NMR covariates.

Participant characteristics are provided in Supplementary Table S1. The GWAS results for the main and secondary analyses are found in Supplementary Table S2. The calculated genomic inflation factors (ranging from 1.003 to 1.010) indicated little evidence of inflation (Supplementary Fig. S1). In EA females and males, n = 30 and n = 54 genome-wide significant (GWS) variants (P < 5e−8) were found, respectively; all were located on chromosome 19 (Supplementary Table S2). In each sex, there was one conditionally independent signal: the top variant was rs56113850 (for C vs. T: beta = 0.67, P = 7.5e−22, MAF = 47.8% in females; beta = 0.75, P = 1.2e−37, MAF = 44.9% in males) (Table 1, Fig. 1, and Supplementary Fig. S2). The rs56113850 variant is found in intron 4 of *CYP2A6* and accounted for 21.8% and 26.1% of rank-transformed NMR variation in EA females and EA males, respectively. The rs56113850 variant was also significant in AA females and AA males (Supplementary Table S2).

**Table 1:** Independent variants from each genome-wide significant locus in EA and AA smokers.

Sample	Chr	Top SNP from region	Imputation Info (Quality) Score	MAF (%)	Effect Allele; Ref. Allele	Beta (SE); P-value	Gene location
EA females (n = 389)	**19**	rs56113850	0.92 in PNAT2	47.8	C; T	0.67 (0.07); 7.5e−22	*CYP2A6* (intron)
EA males (n = 541)	**19**	rs56113850	0.91 in PNAT2	44.9	C; T	0.75 (0.05); 1.2e−37	*CYP2A6* (intron)
AA females (n = 503)	**19**	rs11878604	0.98 in PNAT2, 0.93 in KIS3	22.8	C; T	− 0.71 (0.07); 6.6e−26^c^ − 0.69 (0.11); 5.5e−10 in PNAT2 − 0.73 (0.09); 5.3e−15 in KIS3	*CYP2A6 (*16 kb 3′)
	**19**	19:41352257^d^	0.90 in PNAT2, 0.92 in KIS3	31.9	G; GT	− 0.69 (0.07); 2.1e−25^a,c^ − 0.68 (0.11); 2.9e−9 in PNAT2 − 0.69 (0.08); 2.8e−15 in KIS3	*CYP2A6* (intron)
	***12***	*rs12425845*	*1.0 in PNAT2, 0.99 in KIS3*	*8.4*	*C; T*	*0.64 (0.11); 5.0e−9*^*c*^ *0.73 (0.18); 7.1e−5 in PNAT2* *0.59 (0.14); 2.9e−5 in KIS3*	*TMEM132C (1 Mb 5′)*
AA males (n = 352)	**19**	rs3865454	0.98 in PNAT2, 0.98 in KIS3	34.3	G; T	0.64 (0.07); 1.9e−19^b^ 0.67 (0.09); 1.9e−11 in PNAT2 0.60 (0.11); 1.5e−7 in KIS3	*CYP2A6* (7 kb 3′)
	***6***	*rs9379805*	*1.0 in PNAT2, 0.99 in KIS3*	*33.6*	*A; T*	*− 0.44 (0.08); 4.8e−9*^*b*^ *− 0.34 (0.10); 8.3e−4 in PNAT2* *− 0.58 (0.12); 1.6e−6 in KIS3*	*SLC17A2 (8 kb 5′)*
	***16***	*rs77368288*	*0.81 in PNAT2, 0.73 in KIS3*	*6.5*	*C;T*	*− 0.89 (0.16); 3.5e−8*^*b*^ *− 0.96 (0.18); 4.6e−7 in PNAT2* *− 0.65 (0.34); 5.4e−2 in KIS3*	*ZNF469 (92 kb 5′)*

Novel loci associated with the nicotine metabolite ratio (rank-transformed for analysis) were found in AA females and AA males and are indicated in italics.

*EA* European American, *AA* African American, *Chr* chromosome, *info* info (i.e. quality) score from imputation, *MAF* minor allele frequency, *SE* standard error.

Conditional analysis was used to determine the number of independent signals for each chromosome containing genome-wide significant variants.

^a^After conditioning on rs11878604, the beta (SE) for 19:41352257 was − 0.45 (0.08), and the P-value was 8.3e−9.

^b^Beta, SE, and P-value is reported for the meta-analyzed PNAT2 and KIS3 AA males.

^c^Beta, SE, and P-value is reported for the meta-analyzed PNAT2 and KIS3 AA females.

^d^No rsID is available for this variant in dbSNP.

**Figure 2:**
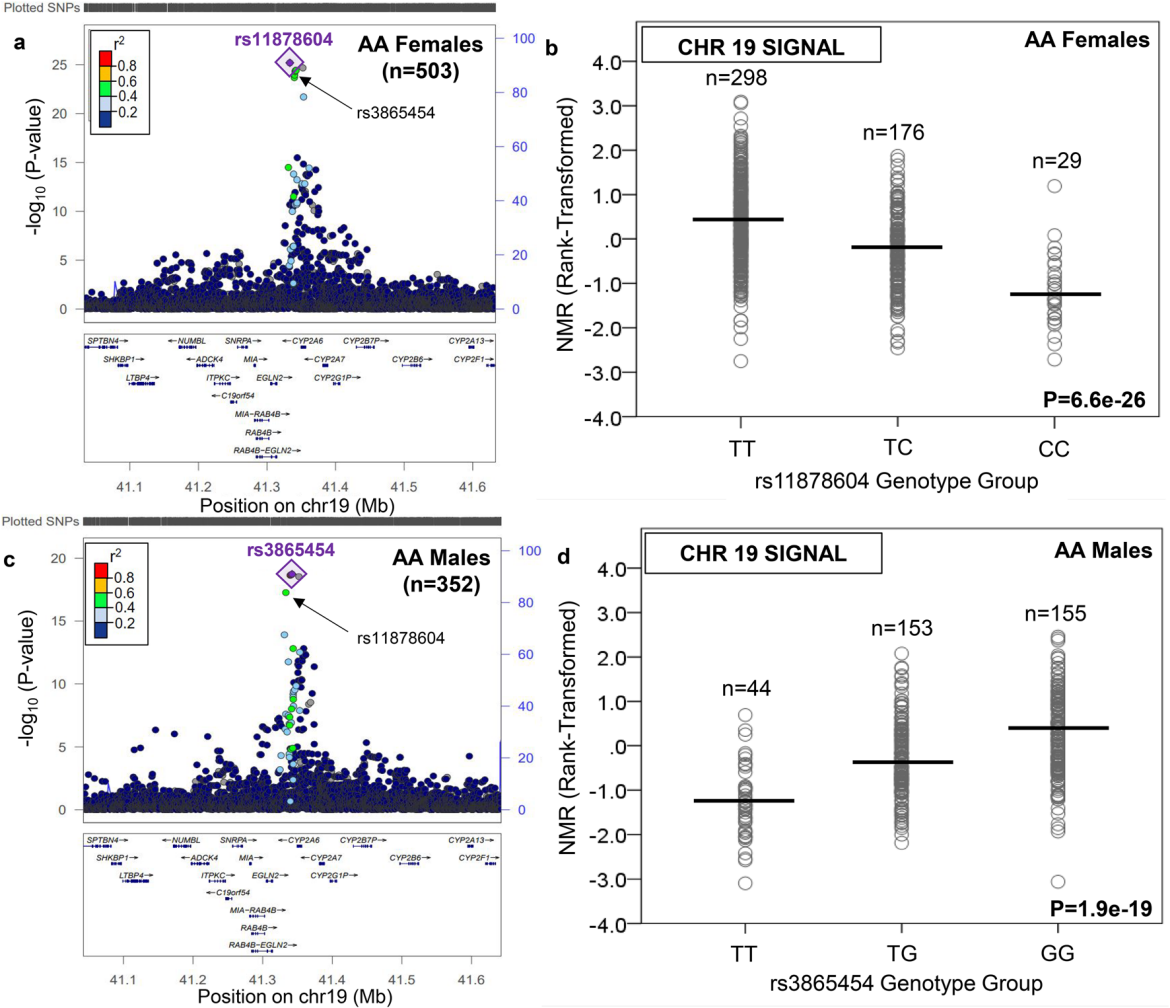
The top signal associated with the Nicotine Metabolite Ratio in African ancestry females and males was found on chromosome 19 in the region containing *CYP2A6*. The nicotine metabolite ratio was rank-transformed for analysis. Regional plots were generated using LocusZoom^44^ (freely available at locuszoom.org). In African American (AA) females, the top variant was rs11878604 (**a**), which was also significant in AA males. The influence of rs11878604 on the Nicotine Metabolite Ratio (NMR) in AA females is shown in (**b**). In AA males, the top variant was rs3865454 (**c**), which was also significant in AA females. The influence of rs3865454 on the NMR in AA males is shown in (**d**). Note: in AA females, there was a second independent signal on chromosome 19: the top variant was an indel at position 41352257 (P = 8.3e−9, after conditioning on rs11878604); this variant was also GWS in AA males. Linkage disequilibrium patterns in (**a**) and (**c**) are based upon the hg19/1000 Genomes November 2014 release African reference population. The plots in (**b**) and (**d**) were created using SPSS version 23 (can be purchased from IBM, Armonk, New York, USA). The black horizontal line represents the mean rank-transformed NMR in each group, and the P-value is from the GWAS after adjusting for population substructure and NMR covariates.

In AA females and males, there were n = 72 and n = 55 GWS variants, respectively (Supplementary Table S2). As for EA, the top overall SNP was found on chromosome 19 in AA. In AA females, the top SNP was rs11878604 (for C vs. T: beta = − 0.71, P = 6.6e−26, MAF = 22.8%) (Table 1, Fig. 2, and Supplementary Fig. S3), located 16 kb 3′ of *CYP2A6*, which explained 16.2% of rank-transformed NMR variation. The rs11878604 variant was the seventh top variant and had a similar beta in AA males, and was also significant in EA males (Supplementary Table S2). There was a second conditionally independent locus on chromosome 19 in AA females, with the top variant found at position 41352257 (for G vs. GT: beta = − 0.69, P = 2.2e−25, MAF = 31.9%) located in an intron of *CYP2A6* (Table 1); this variant explained 3.6% of rank-transformed NMR variation after controlling for rs11878604, and was the sixth top variant and had a similar beta in AA males (Supplementary Table S2). In AA males, a single independent locus on chromosome 19 was observed. The top overall SNP was rs3865454 (for G vs. T: beta = 0.64, P = 1.9e−19, MAF = 34.3%) (Table 1, Fig. 2, and Supplementary Fig. S3), located 7 kb 3′ of *CYP2A6*, which explained 18.9% of rank-transformed NMR variation. The rs3865454 variant was the fourth top variant and had a similar beta in AA females (Supplementary Table S2). The betas for the top variants (rs11878604, variant at position 41352257, and rs3865454) were similar between PNAT2 AA and KIS3 AA (Supplementary Table S2). Of note, the top chromosome 19 SNPs (i.e. rs56113850 in EA males and EA females, rs11878604 in AA females, and rs3865454 in AA males) are in low-moderate linkage disequilibrium (LD; r^2^ = 0.13–0.51) in AA and EA populations (Supplementary Fig. S4).

**Figure 3:**
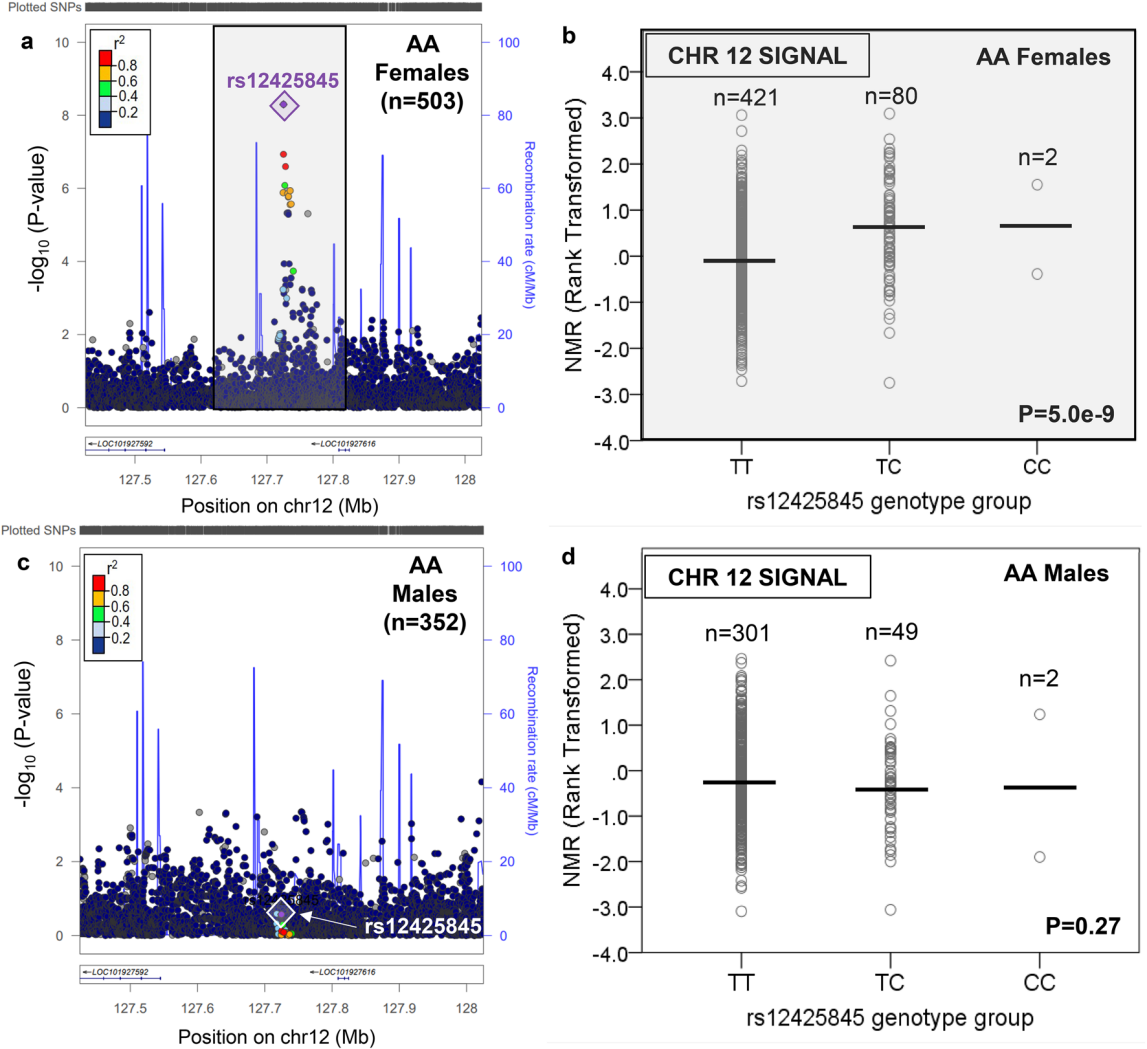
There was a chromosome 12 signal associated with the Nicotine Metabolite Ratio in African American females which was not significant in African American males. The nicotine metabolite ratio was rank-transformed for analysis. A regional plot, generated using LocusZoom^44^ (freely available at locuszoom.org), shows the top overall chromosome 12 variant (rs12425845) in AA females (**a**); the influence of rs12425845 on the Nicotine Metabolite Ratio (NMR) in AA females is shown in (**b**). The chromosome 12 locus containing rs12425845 was not significant in AA males (**c**); the lack of influence of rs12425845 on the NMR in AA males is shown in (**d**). Linkage disequilibrium patterns in (**a**) and (**c**) are based upon the hg19/1000 Genomes November 2014 release African reference population. The plots in (**b**) and (**d**) were created using SPSS version 23 (can be purchased from IBM, Armonk, New York, USA). The black horizontal line represents the mean rank-transformed NMR in each group, and the P-value is from the GWAS after adjusting for population substructure and NMR covariates.

**Figure 4:**
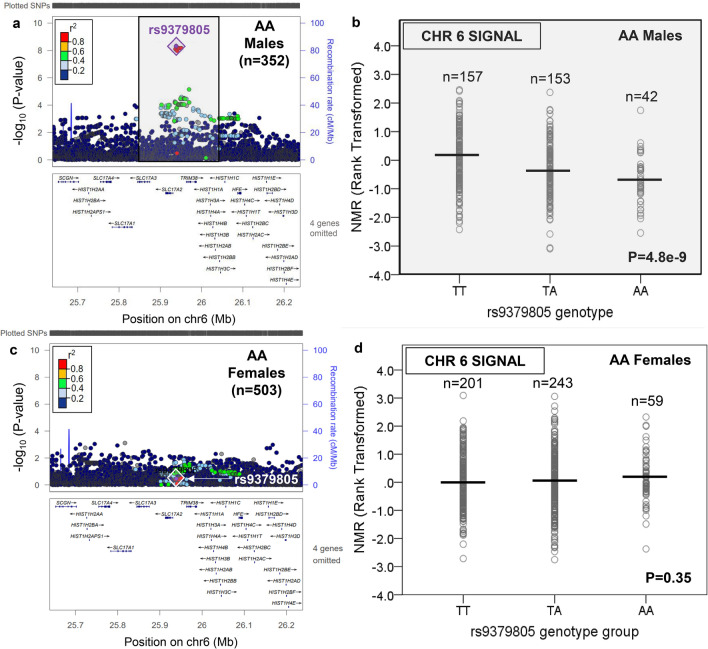
There was a chromosome 6 signal associated with the Nicotine Metabolite Ratio in African American males which was not significant in African American females. The nicotine metabolite ratio was rank-transformed for analysis. A regional plot, generated using LocusZoom^44^ (freely available at locuszoom.org), shows the top overall chromosome 6 variant (rs9379805) in AA males (a); the influence of rs9379805 on the Nicotine Metabolite Ratio (NMR) in AA males is shown in (**b**). The chromosome 6 locus containing rs9379805 was not significant in AA females (c); the lack of influence of rs9379805 on the NMR in AA females is shown in (**d**). Linkage disequilibrium patterns in (**a**) and (**c**) are based upon the hg19/1000 Genomes November 2014 release African reference population. The plots in (**b**) and (**d**) were created using SPSS version 23 (can be purchased from IBM, Armonk, New York, USA). The black horizontal line represents the mean rank-transformed NMR in each group, and the P-value is from the GWAS after adjusting for population substructure and NMR covariates.

**Figure 5:**
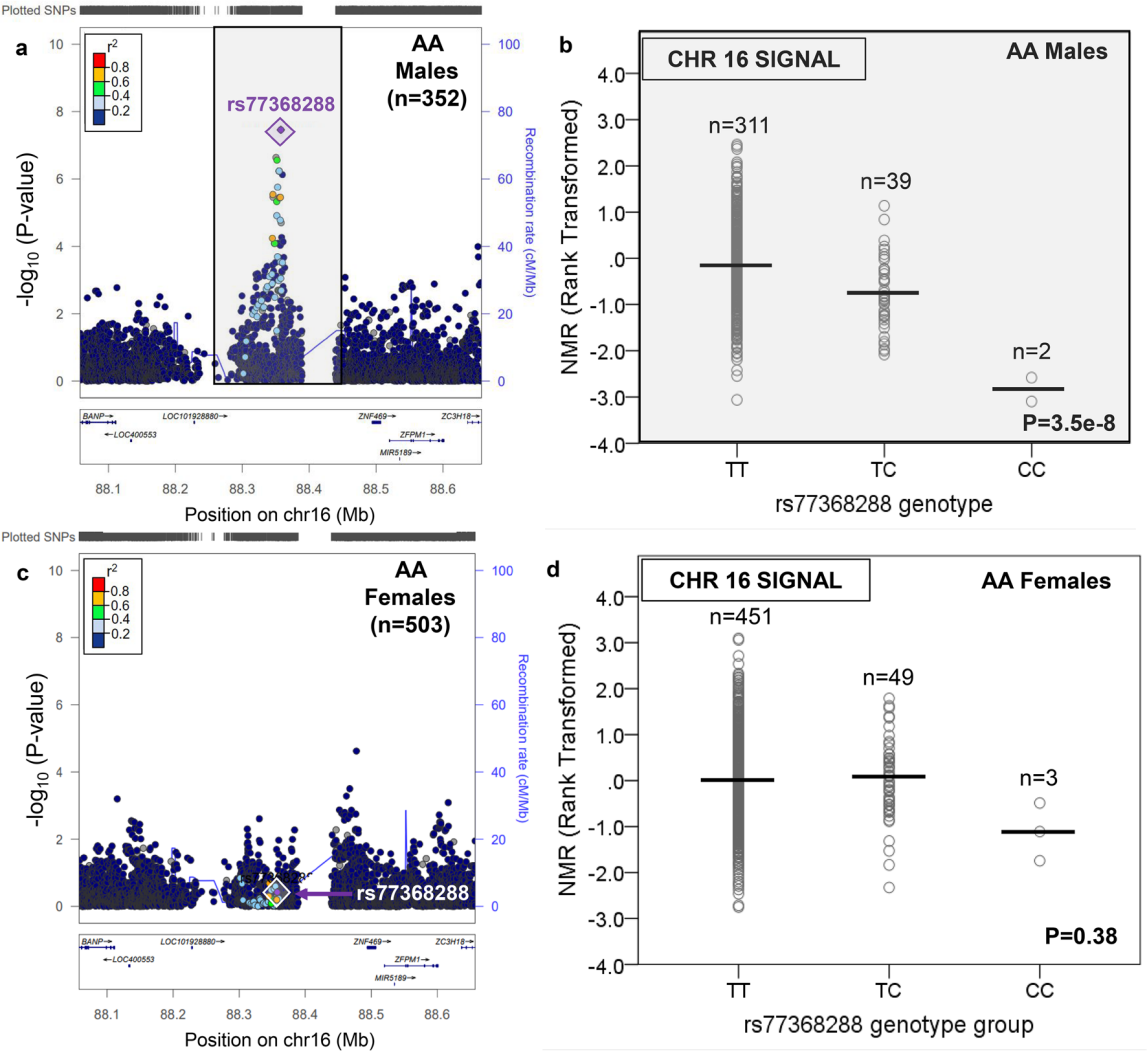
There was a chromosome 16 signal associated with the Nicotine Metabolite Ratio in African American males which was not significant in African American females. The nicotine metabolite ratio was rank-transformed for analysis. A regional plot, generated using LocusZoom LocusZoom^44^ (freely available at locuszoom.org), shows the top overall chromosome 16 variant (rs77368288) in AA males (**a**); the influence of rs77368288 on the Nicotine Metabolite Ratio (NMR) in AA males is shown in (**b**). The chromosome 16 locus containing rs77368288 was not significant in AA females (**c**); the lack of influence of rs77368288 on the NMR in AA females is shown in (**d**). Linkage disequilibrium patterns in (**a**) and (**c**) are based upon the hg19/1000 Genomes November 2014 release African reference population. The plots in (**b**) and (**d**) were created using SPSS version 23 (can be purchased from IBM, Armonk, New York, USA). The black horizontal line represents the mean rank-transformed NMR in each group, and the P-value is from the GWAS after adjusting for population substructure and NMR covariates.

In contrast to EA, there were novel GWS loci found outside of chromosome 19 in AA (Supplementary Table S2). In AA females, one additional locus was found, on chromosome 12 (Table 1, Fig. 3, and Supplementary Fig. S5). The chromosome 12 signal comprised one GWS SNP, rs12425845 (for C vs. T: beta = 0.64, P = 5.0e−9, MAF = 8.4%), located ~ 1 MB 5′ of *TMEM132C*, which explained 6.1% of rank-transformed NMR variation. This chromosome 12 locus was not GWS in AA males (Fig. 3). In AA males, two additional loci were found outside of chromosome 19, on chromosome 6 (six GWS SNPs representing one independent signal) and chromosome 16 (one GWS SNP) (Table 1 and Figs. 4, 5 and Supplementary Fig. S5). The top chromosome 6 GWS SNP was rs9379805 (for A vs. T: beta = − 0.44, P = 4.8e−9, MAF = 33.6%), located ~ 8 kb 5′ of *SLC17A2*, which explained 8.0% of rank-transformed NMR variation. The lone chromosome 16 GWS SNP, rs77368288 (for C vs. T: beta = − 0.89, P = 3.5e−8, MAF = 6.5%), was found ~ 93 kb 5′ of *ZNF469* and explained 7.1% of rank-transformed NMR variation. Neither the chromosome 6 nor the chromosome 16 loci were GWS in AA females (Figs. 4, 5). In AA, a significant sex × SNP interaction effect on rank-transformed NMR was observed for all three novel loci outside of chromosome 19: rs12425845 (P = 0.00002), rs9379805 (P = 0.000002), and rs77368288 (P = 0.001). Notably, variation in *MRGPRX4*, previously associated with menthol smoking in AA^45^, was not associated with rank-transformed NMR in either AA males or AA females (Supplementary Fig. S6).

In all secondary analyses, we observed similar effect sizes (betas) to those found in the main analysis with few substantial differences (Supplementary Table S2). Overall, the betas and P-values from the secondary analyses were highly correlated with the betas and P-values, respectively, from the main analysis (Supplementary Table S3).

### Replication analysis

None of the signals from chromosome 12 (in AA females), chromosome 6 (in AA males), or chromosome 16 (in AA males) were significantly associated with the NMR in external clinical trials (i.e. KIS2 and Quit-2-Live), however the direction of effect for the chromosome 12 and 16 signals seemed consistent (Supplementary Fig. S7). Of note, the chromosome 12 and 6 signals were associated with the NMR in both PNAT2 (P = 7.1e−5 and 8.2e−4, respectively) and KIS3 (P = 2.9e−5 and P = 1.6e−6, respectively) (Table 1 and Supplementary Table S2). The chromosome 19 locus that we identified is consistent with prior GWASs of the NMR^24–28^.

### eQTL and meQTL analyses

Most of the GWS variants associated with the NMR were significant eQTL and/or meQTL (Supplementary Table S2). In EA, the top SNP (rs56113850) was significantly associated with altered expression of CYP2A6, CYP2A7, EGLN2, RAB4B, CYP2G1P, CYP2T1P, and CTC-490E21.11, as previously noted^27^. In EA males, additional GWS variants mapping to *CYP2A6* and *CYP2A7* were associated with altered CYP2A6 expression in GTEx in the adrenal gland, which were not GWS in females (Supplementary Table S2).

In AA females, the top chromosome 19 SNP (rs11878604) was a significant eQTL for CYP2A6 in the adrenal gland, as well as for CYP2G1P, but was not a significant meQTL (Supplementary Table S2). In AA males, the top chromosome 19 SNP (rs3865454) was significantly associated with altered expression of CYP2A7, CYP2T1P, C19orf54, and CTC-490E21.11. However, rs3865454 was not a significant cis or trans meQTL. There were also several GWS SNPs in chromosome 19 in AA that were significant eQTL for CYP2A6 in subcutaneous adipose tissue.

The GWS SNP from chromosome 12 found in AA females (rs12425845) was a significant trans meQTL influencing methylation at a CpG site on chromosome 12 within *TMEM132A* but was not a significant eQTL. The top chromosome 6 SNP found in AA males (rs9379805), was not found in GTEx, however other SNPs from this locus were significant eQTL for several targets including three drug transporters (i.e. SLC17A1, SLC17A2, and SLC17A3) in adrenal gland and/or testis (Supplementary Table S2). Moreover, the GWS SNPs from the chromosome 6 locus were all cis meQTL. The GWS SNP from chromosome 16 in AA males (rs77368288) was a significant eQTL for APRT involved in the nucleotide salvage pathway.

## Discussion

This is the first genome-wide investigation of sex differences in the genetic influences on the NMR, a sexually dimorphic nicotine metabolism biomarker^5,6^. Smoking behaviours, cessation outcomes, and tobacco-related disease risk, which are each associated with the NMR^15,17,46^, also differ between men and women^2,47–49^. We further stratified our sex-based analyses by genetic ancestry, due to differences in population substructure as well as in the NMR; AA have lower NMR on average compared to EA^8^. Moreover, smoking cessation outcomes^39^ and lung cancer risk^50^ differ between EA and AA.

In all four analytic sub-groups, the top overall variant was found on chromosome 19 and mapped to the *CYP2A6* gene coding for the main metabolic enzyme for nicotine. In EA females and EA males, a single GWS locus was found on chromosome 19: the top variant was rs56113850 (intron 4 of *CYP2A6*). No other chromosomes contained GWS loci. The rs56113850 variant was the top SNP in all prior GWASs of the NMR in EA smokers that controlled for sex^24,25,27,28^ and was also significant in AA. Chromosome 19 also contained the top SNP in AA females (rs11878604, located 16 kb 3′ of *CYP2A6*) and AA males (rs3865454; 7 kb 3′ of *CYP2A6*). The rs11878604 and rs3865454 variants are in moderate LD (r^2^ = 0.48) in AA populations, and the rs11878604 variant was found in our prior NMR GWAS in AA (controlled for sex)^26^. The rs11878604 variant was also GWS in EA males, while rs3865454 was not significant in EA. The difference in top variant between EA and AA is likely due in part to distinct LD patterns and variant frequencies. In AA, rs28399454, which defines the known reduced function CYP2A6*17 allele^51^, was also GWS; rs28399454 is in low LD with rs11878604 (r^2^ = 0.28) and rs3865454 (r^2^ = 0.21). In contrast to EA, significant and novel loci were found outside of chromosome 19 in AA; these loci differed between AA females (chromosome 12) and AA males (chromosomes 6 and 16). In AA, the additional, and sex-specific, loci in our current study underscore the potential importance of performing sex-based analyses to better understand the biology of sexually dimorphic traits^29^ such as the NMR.

In the GWAS catalog (https://www.ebi.ac.uk/gwas/; accessed June 30, 2021), there are no reported associations for rs12425845 (chromosome 12), rs9379805 (chromosome 6), or rs77368288 (chromosome 16) with any traits. Only rs9379805 was found within 10 kb of a gene (i.e. *SLC17A2*). The SLC17A1-4 transporters are thought to transport organic anions, however their substrates and functional relevance are only partly understood^52^. Whether cotinine and/or 3′hydroxycotinine are substrates of the SLC17 transporter family is not known. The mechanism explaining the association between each of these non-chromosome 19 regions and the NMR remains to be determined but may involve regulatory regions, as an example.

Our study had several limitations, including the lack of association of the NMR with the sex-specific signals on chromosomes 12, 6, and 16 in an external sample, despite being sufficiently powered. In the discovery cohort, the chromosome 12 and 16 signals comprised a single GWS SNP, although other SNPs with low P-values in LD with the GWS SNP were found in the region. The inclusion criteria and recruitment strategy also differed between trials. However, in AA, the betas for the top SNPs were similar between PNAT2 and KIS3 despite differences in baseline smoking level. Further testing in external datasets is required to determine the robustness of the sex-specific findings. In our analyses controlling for menthol, many P-values were less significant than in models not controlling for menthol, however this could be due to the smaller sample size as the betas were highly correlated. Both GTEx and mQTLdb contain data from predominantly European (85% and 100%, respectively) samples^43^, limiting their generalizability to AA. In GTEx, 67% of the donors are male and sex differences in transcript levels have recently been identified^53^. Future studies could assess tissue expression levels from male and female donors separately. Moreover, all of the adults contributing samples to the mQTLdb are female, limiting generalizability to males; we therefore included all time-points in the mQTLdb so that multiple sexes could be considered. Finally, we examined the autosomes in this paper; examination of the X chromosome, which requires unique quality control procedures and statistical modeling considerations^54^, is underway. Strengths of our study include the sex-based analyses and the examination of multiple ancestries. Our sub-group analyses were well-powered for common variants (MAF ≥ 5%) each explaining at least 7–10% of rank-transformed NMR variance. The top SNPs each explained a relatively large portion of the variance in the NMR, consistent with previous NMR GWASs^24,26–28^. Future sex-based ancestry-specific analyses of the NMR, including genome-wide testing for sex-by-genotype interactions, in larger samples will determine the robustness of our findings, reveal new loci, and may suggest utility of sex-based genetic risk scoring approaches for the NMR.

In conclusion, we showed potential sex differences in the genetic influences on the NMR in AA, but not EA smokers. Identifying the causal SNPs and the mechanism behind these sex-based genetic influences will provide further biological insight into the NMR, which differs by sex^8^ and can only be measured in current tobacco users^22^. A greater understanding of how sex and ancestry influence nicotine metabolism biomarker genetics may improve genomics-based risk prediction modelling for tobacco-related illnesses and enhance personalized treatment approaches for tobacco dependence.

## Supplementary Information

Supplementary Information.

Supplementary Table S2.

## Data Availability

Due to the type of informed consents given by study participants, individual level data cannot be made publicly available. GWAS summary statistics and scripts used for analyses are available upon request.
