# Supplementary material for: Analyses of nicotine metabolism biomarker genetics stratified by sex in African and European Americans
Source: Sci Rep. 2021 Oct 1;11:19572. doi: 10.1038/s41598-021-98883-z (PMC8486765; doi:10.1038/s41598-021-98883-z)
Supplement: Supplementary file 1 — Supplementary Information. [file 41598_2021_98883_MOESM1_ESM.docx]

**SUPPLEMENTARY INFORMATION**

**Analyses of nicotine metabolism biomarker genetics stratified by sex in African and European Americans**

**Authors**

Meghan J. Chenoweth, PhD^1,2^, Lisa Sanderson Cox, PhD^3^, Nikki L. Nollen, PhD^3^, Jasjit S. Ahluwalia, MD, MPH^4^, Neal L. Benowitz, MD^5^, Caryn Lerman, PhD^6^, Jo Knight, PhD^7,8^, Rachel F. Tyndale, PhD^1,2,8^

**Affiliations**

^1^Campbell Family Mental Health Research Institute, Centre for Addiction and Mental Health, Toronto, Ontario, Canada

^2^Department of Pharmacology and Toxicology, University of Toronto, Toronto, Ontario, Canada

^3^Department of Population Health, University of Kansas School of Medicine, Kansas City, Kansas, USA

^4^Departments of Behavioral and Social Sciences and Medicine, Brown University, Providence, Rhode Island, USA

^5^Department of Medicine, University of California, San Francisco, San Francisco, California, USA

^6^USC Norris Comprehensive Cancer Center, Keck School of Medicine, University of Southern California, Los Angeles, California, USA

^7^Data Science Institute and Lancaster University Medical School, Lancaster, UK

^8^Department of Psychiatry, University of Toronto, Toronto, Ontario, Canada

**Table S1.** Study participant characteristics assessed at baseline in the PNAT2 and KIS3 clinical trials

| Characteristic | EA Females  (PNAT2)  n=389 | EA Males  (PNAT2)  n=541 | AA Females  (PNAT2)  n=205 | AA Males  (PNAT2)  n=201 | AA Females  (KIS3)  n=298 | AA Males  (KIS3)  n=151 |
| --- | --- | --- | --- | --- | --- | --- |
| Age (years), mean (SD); range | 47 (11),  20 - 65 | 46 (11);  18 - 65 | 48 (10);  23 - 65 | 47 (10);  20 - 64 | 46 (12);  19 - 80 | 49 (10);  26 - 75 |
| BMI (kg/m^2^), mean (SD); range | 27.8 (6.3);  17.2 – 53.2 | 28.3 (5.4);  17.1 – 50.0 | 33.1 (7.6);  17.6 – 58.3 | 28.4 (6.0);  18.3 – 51.7 | 31.9 (8.1);  14.8 – 62.8 | 29.7 (7.2);  18.1 – 68.4 |
| Cigarettes/day, mean (SD); range | 19 (8);  8 - 75 | 21 (7);  6 - 50 | 16 (5);  10 - 35 | 17 (7);  5 - 40 | 8 (3);  1 - 17 | 8 (3);  1 - 15 |
| Cotinine (ng/ml), mean (SD), median;  range | 238 (109),  216;  37 – 667 | 244 (105),  226;  42 – 911 | 286 (123),  261;  69 – 802 | 278 (141),  252;  32 – 837 | 247 (119),  239;  16 – 681 | 238 (130),  218;  14 – 606 |
| NMR,  mean (SD), median;  range | 0.44 (0.21),  0.41;  0.02 – 1.35 | 0.39 (0.19),  0.36;  0.01 – 1.39 | 0.32 (0.20),  0.27;  0.03 – 1.17 | 0.28 (0.19),  0.23;  0.01 – 1.15 | 0.40 (0.27),  0.35;  0.02 – 1.79 | 0.35 (0.23),  0.29;  0.02 – 1.52 |

Abbreviations: PNAT, Pharmacogenetics of Nicotine Addiction Treatment; KIS, Kick-It-At-Swope; EA, European American; AA, African American; SD, standard deviation; BMI, body mass index; NMR, nicotine metabolite ratio. Note: After conducting sex-specific GWAS analyses in PNAT2 and KIS3, the PNAT2 and KIS3 AA females were meta-analyzed, as were the PNAT2 and KIS3 AA males.

**Table S3. Correlations between the effect sizes (betas), and correlations between the P-values, for all genome-wide significant variants in the main analyses compared to the secondary analyses.**

| Secondary Analysis | Group analyzed | | Females: Correlation between  GWAS betas, and between GWAS P-values | Males: Correlation between  GWAS betas, and between GWAS P-values |
| --- | --- | --- | --- | --- |
| Controlling for menthol | | PNAT2 EA females and PNAT2 EA males^a^ | GWAS betas: Rho=0.90; P=1.34e-11  GWAS P-values: Rho=0.91; P=3.60e-12 | GWAS betas: Rho=0.98; P=8.12e-36  GWAS P-values: Rho=0.94; P=4.38e-26 |
| Controlling for alcohol | | KIS3 AA females and KIS3 AA males^b^ | GWAS betas: Rho=0.99; P=7.44e-67  GWAS P-values: Rho=0.98; P=1.71e-49 | GWAS betas: Rho=1.0; P=4.48e-81  GWAS P-values: Rho=1.0; P=4.48e-81 |
| Not controlling for menthol | | All AA females and  all AA males^c^ | GWAS betas: Rho=1.0; P=1.97e-72  GWAS P-values: Rho=0.97; P=6.63e-47 | GWAS betas: Rho=0.99; P=1.84e-51  GWAS P-values: Rho=0.94; P=1.30e-26 |

Abbreviations: PNAT, Pharmacogenetics of Nicotine Addiction Treatment; KIS, Kick-It-At-Swope; EA, European American; AA, African American.

^a^The sample size for this secondary analysis (i.e. n=239 females, n=351 males) was smaller than the main analysis (i.e. n=389 females, n=541 males) due to missing menthol data in the secondary analysis.

^b^The sample size for this secondary analysis was the same as the main analysis (i.e. n=298 females, n=151 males).

^c^The sample size for this secondary analysis (i.e. n=552 females, n=401 males) was higher than the main analysis (i.e. n=503 females, n=352 males) due to missing menthol data in the main analysis.

**
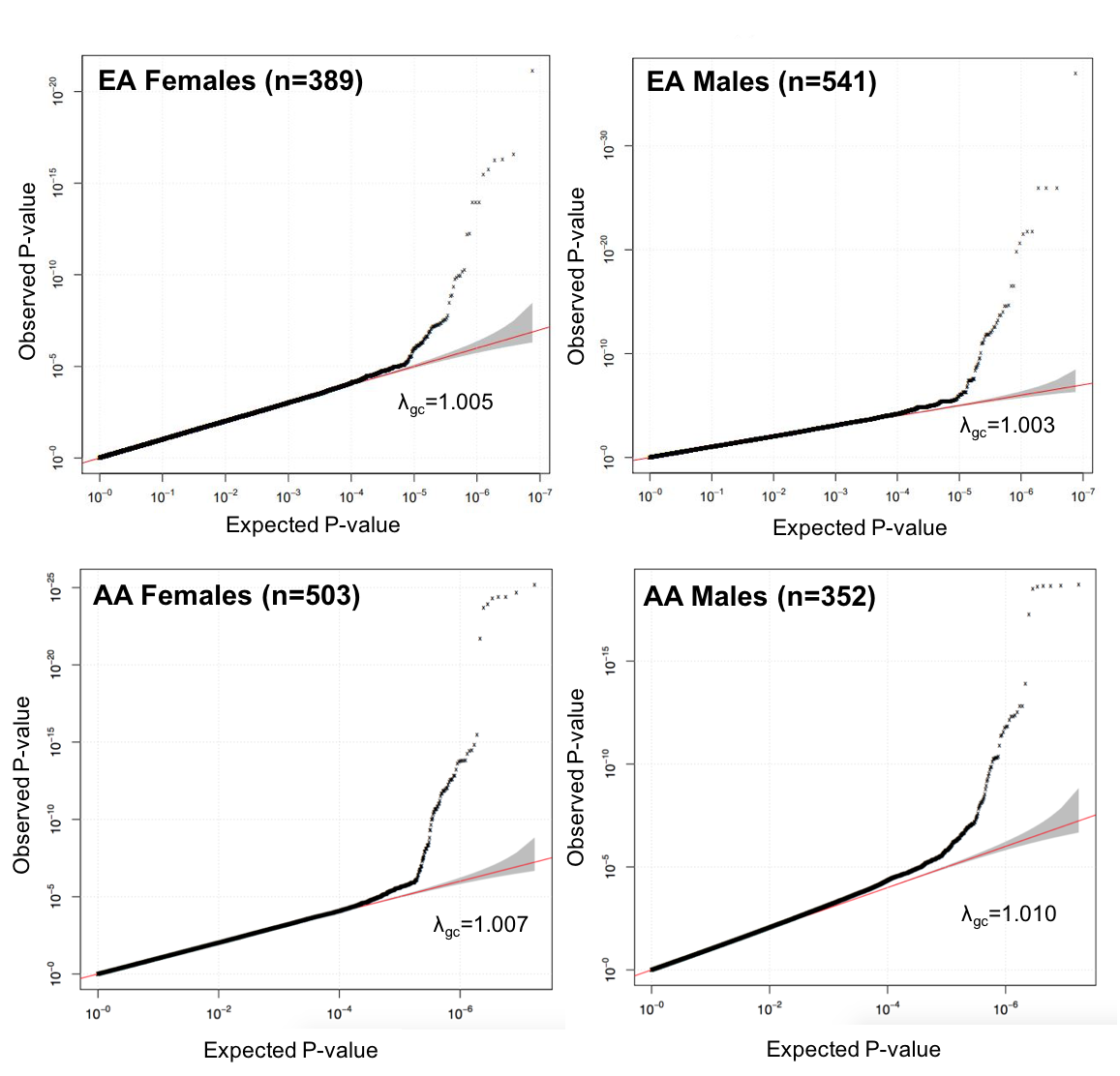
**

**Figure S1. Quantile-Quantile (QQ) plots depicting the expected P-value against the observed P-value in the sex- and ancestry-stratified GWAS of the nicotine metabolite ratio.** The nicotine metabolite ratio was rank-transformed for analysis. QQ plots are shown separately for European American (EA) females, EA males, African American (AA) females, and AA males. The genomic inflation factor, λ_gc_, is indicated on the plots. Analyses included genotyped and imputed variants, with minor allele frequencies >1% and imputation info scores > 0.60. Plots were created using R version 3.2.1.

**Figure S2. The top variant associated with the Nicotine Metabolite Ratio in European ancestry females and males was r56113850.** The influence of rs56113850 on the raw (i.e. untransformed) nicotine metabolite ratio (NMR) is shown in EA females **(A)** and EA males **(B).** The black horizontal line represents the mean NMR in each group. In EA females, the mean NMR was 0.31, 0.43, and 0.57 in the rs56113850 TT, CT, and CC genotype groups, respectively. In EA males, the mean NMR was 0.26, 0.37, and 0.50 in the rs56113850 TT, CT, and CC genotype groups, respectively. The plots were created using SPSS version 23 (can be purchased from IBM, Armonk, New York, USA).


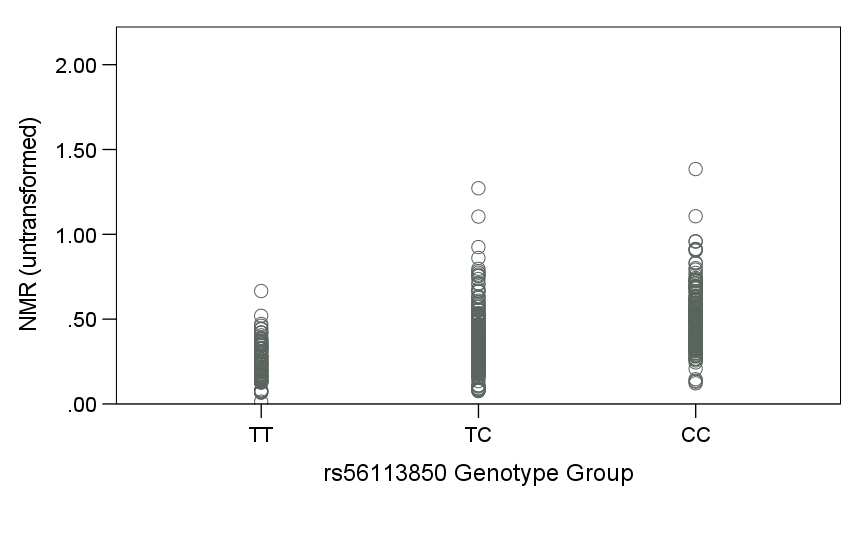

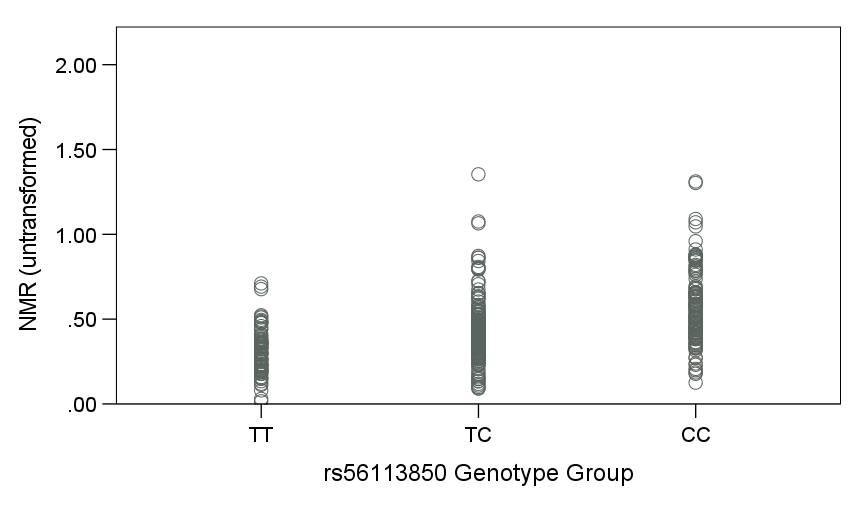


**CHR 19 SIGNAL**

n=89

n=181

n=119

**CHR 19 SIGNAL**

**EA Males**

n=101

n=259

n=181

**A)**

**B)**

**EA Females**

**Figure S3. The top variant associated with the Nicotine Metabolite Ratio in African ancestry females and males was rs11878604 and rs3865454, respectively.** The influence of rs11878604 on the raw (i.e. untransformed) nicotine metabolite ratio (NMR) is shown in AA females in **(A)**. The influence of rs3865454 on the raw (i.e. untransformed) NMR is shown in AA males in **(B)**. The black horizontal line represents the mean NMR in each group. In AA females, the mean NMR was 0.43, 0.29, and 0.15 in the rs11878604 TT, CT, and CC genotype groups, respectively. In AA males, the mean NMR was 0.14, 0.28, and 0.39 in the rs3865454 TT, TG, and GG genotype groups, respectively. The plots were created using SPSS version 23 (can be purchased from IBM, Armonk, New York, USA).


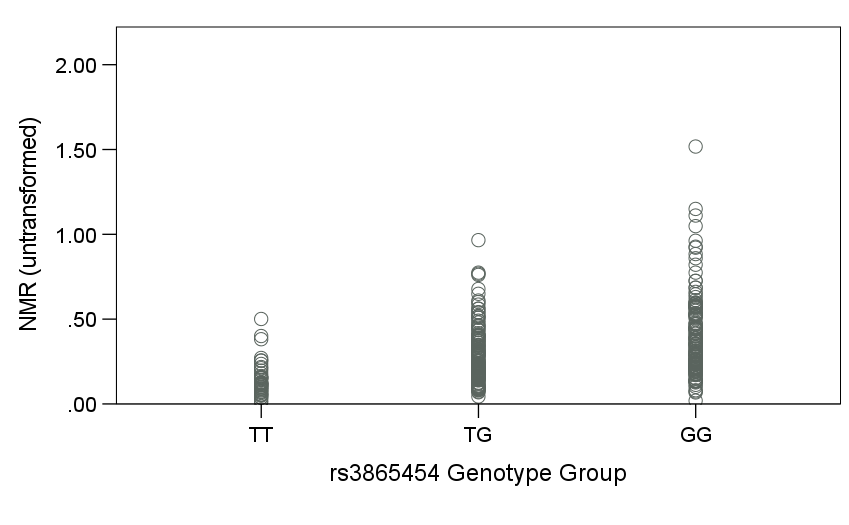

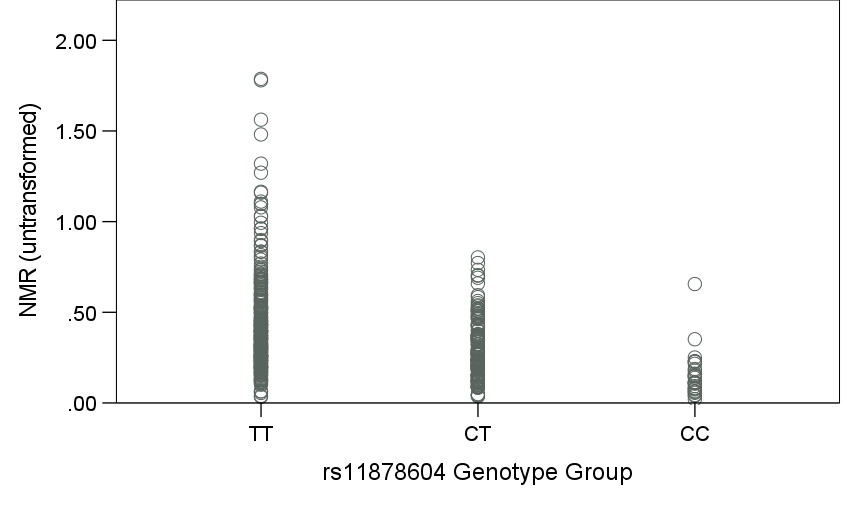


**CHR 19 SIGNAL**

**A)**

**AA Females**

n=298

n=176

n=29

**CHR 19 SIGNAL**

**AA Males**

**B)**

n=44

n=153

n=155

**
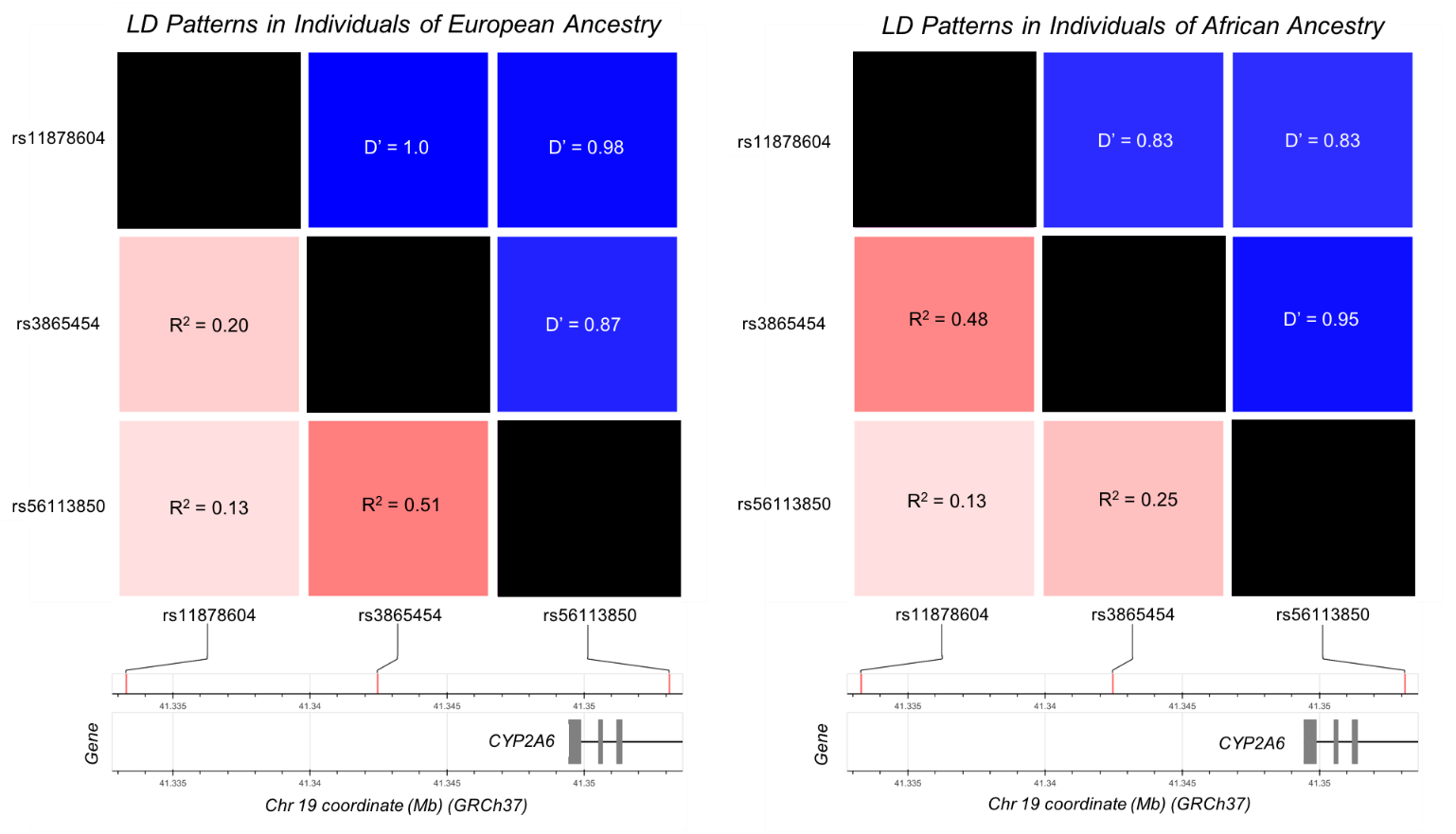
**

**Figure S4. Linkage Disequilibrium (LD) relationships for the top chromosome 19 variants found in the sex- and ancestry-stratified GWAS of the nicotine metabolite ratio.** The nicotine metabolite ratio was rank-transformed for analysis. LD relationships (R^2^ and D’ values) between rs56113850 (top variant in EA females and in EA males), rs11878604 (top variant in AA females), and rs3865454 (top variant in AA males) were calculated separately in individuals of EA ancestry and AA ancestry using the LDmatrix Tool in the freely available LDlink suite of web-based applications (<https://ldlink.nci.nih.gov/)>. For the LD analysis in EA, all European populations were selected in LDlink. For the LD analysis in AA, all African populations were selected in LDlink. In both ancestral groups, the three top variants are in low-moderate LD, as indicated by the R^2^ values ranging from 0.13 to 0.51 in EA, and from 0.13 to 0.48 in AA.

**
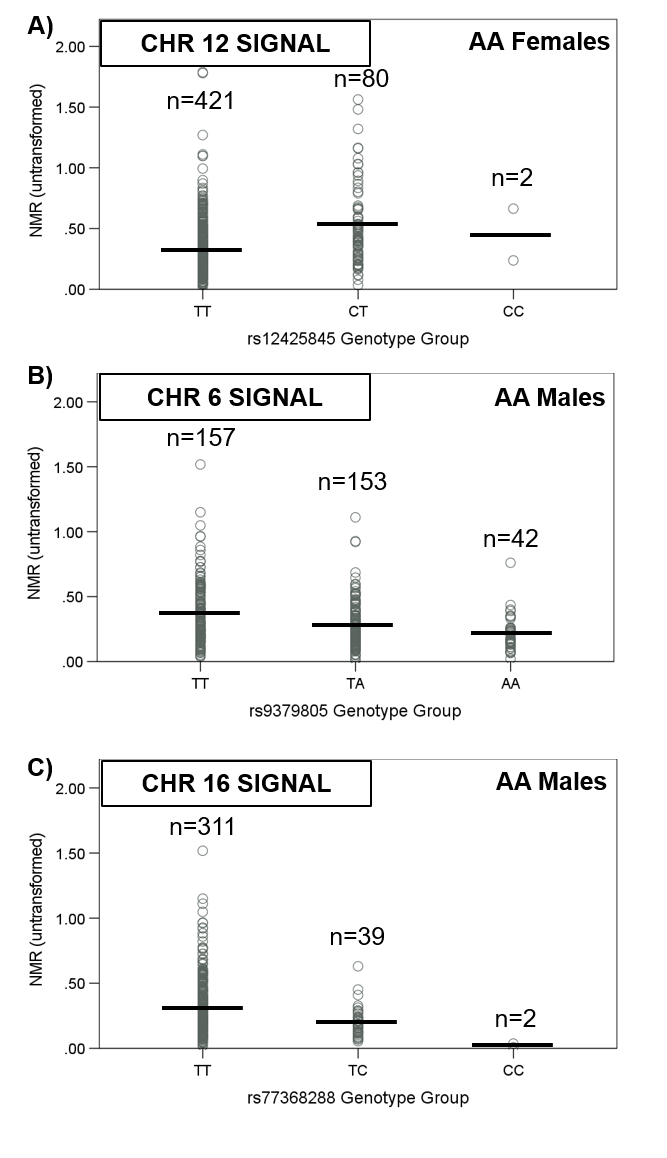
**

**Figure S5. Outside of chromosome 19, there was a significant locus on chromosome 12 in African ancestry females (top variant: rs12425845), and on chromosomes 6 (top variant: rs9379805) and 16 (top variant: rs77368288) in African ancestry males.** The influence of rs12425845 on the raw (i.e. untransformed) nicotine metabolite ratio (NMR) is shown in AA females in **(A)**. The influence of rs9379805 on the raw (i.e. untransformed) NMR is shown in AA males in **(B)**. The influence of rs77368288 on the raw (i.e. untransformed) NMR is shown in AA males in **(C)**. The black horizontal line represents the mean NMR in each group. In AA females, the mean NMR was 0.34, 0.52, and 0.45 in the rs12425845 TT, CT, and CC genotype groups, respectively. In AA males, the mean NMR was 0.37, 0.27, and 0.21 in the rs9379805 TT, TA, and AA genotype groups, respectively. In AA males, the mean NMR was 0.33, 0.20, and 0.02 in the rs77368288 TT, TC, and CC genotype groups, respectively. The plots were created using SPSS version 23 (can be purchased from IBM, Armonk, New York, USA).


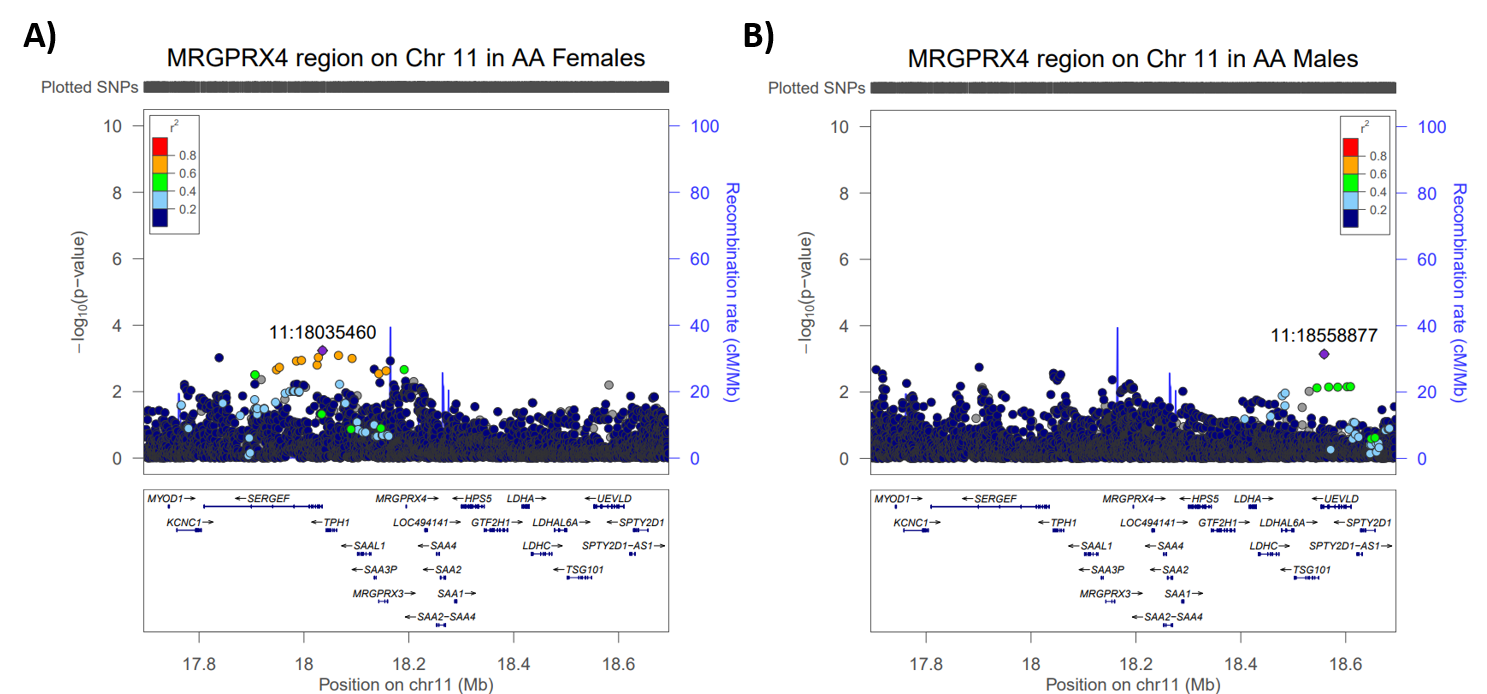


**Figure S6. Variation in *MRGPRX4*, on chromosome 11, was not associated with the Nicotine Metabolite Ratio in African ancestry females and males.** The nicotine metabolite ratio was rank-transformed for analysis. *MRGPRX4* did not reach genome-wide significance in either AA females **(A)** or AA males **(B)**. Linkage disequilibrium patterns are based upon the hg19/1000 Genomes November 2014 release African reference population. The plots were generated using LocusZoom (44) (freely available at locuszoom.org).

**
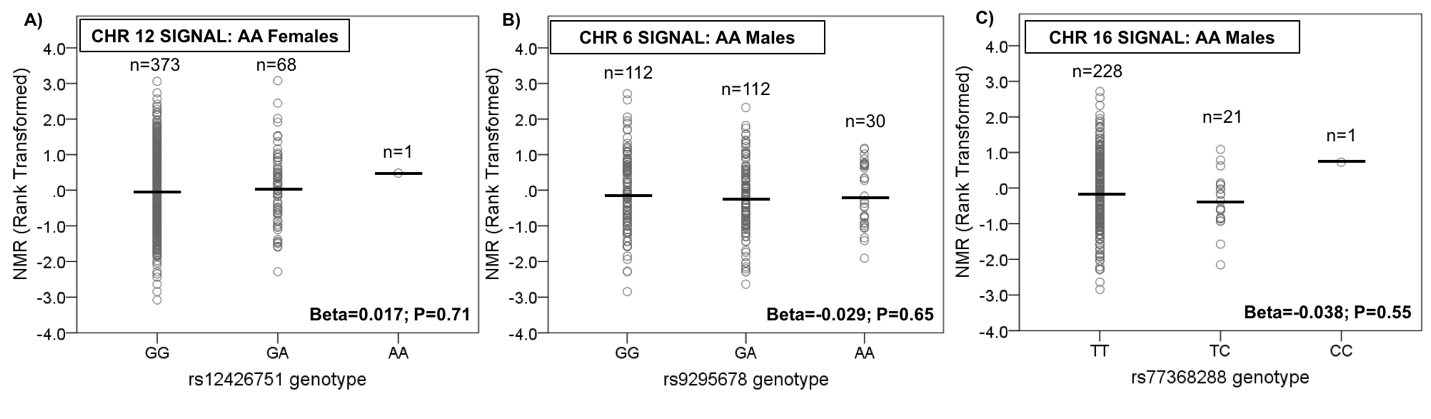
**

**Figure S7. The chromosome 12 signal in African American females, and the chromosome 6 and 16 signals in African American males, did not replicate in smokers pooled from two external clinical trials, Kick-it-at-Swope (KIS)-2 and Quit-2-Live (Q2L).** The nicotine metabolite ratio (NMR; rank-transformed) is plotted against a proxy SNP (i.e. rs12426751; in high linkage disequilibrium (r^2^ = 0.91) with the top SNP rs12425845) for the chromosome 12 signal in KIS2 + Q2L African American (AA) females **A)**, and against a proxy SNP (i.e. rs9295678; in high linkage disequilibrium (r^2^ = 0.95) with the top SNP rs9379805) for the chromosome 6 signal in KIS2 + Q2L AA males **B)**. The nicotine metabolite ratio (rank-transformed) is plotted against the top chromosome 16 SNP (rs77368288) in KIS2 + Q2L AA males **C)**. The standardized betas and P-values indicated are from linear regression models examining the influence of the SNP (coded additively) on rank-transformed NMR after controlling for age, BMI, and menthol cigarette smoking. The black horizontal line represents the mean rank-transformed NMR in each group. The plots were created using SPSS version 23 (can be purchased from IBM, Armonk, New York, USA).
